# MET inhibition enhances PARP inhibitor efficacy in castration‐resistant prostate cancer by suppressing the ATM/ATR and PI3K/AKT pathways

**DOI:** 10.1111/jcmm.17037

**Published:** 2021-11-10

**Authors:** Sihai Zhou, Zhihong Dai, Liang Wang, Xiang Gao, Liqin Yang, Zhenwei Wang, Qi Wang, Zhiyu Liu

**Affiliations:** ^1^ Department of Urology The Second Affiliated Hospital of Dalian Medical University DaLian China; ^2^ Department of Urology Guangdong Second Provincial General Hospital Guangzhou China; ^3^ Department of Respiratory Medicine The Second Affiliated Hospital of Dalian Medical University DaLian China

**Keywords:** ATM/ATR pathway, CRPC, DNA damage response, MET inhibitor, PARP inhibitor, PI3K/AKT pathway

## Abstract

Up to 30% of patients with metastatic castration‐resistant prostate cancer (CRPC) patients carry altered DNA damage response genes, enabling the use of poly adenosine diphosphate–ribose polymerase (PARP) inhibitors in advanced CRPC. The proto‐oncogene mesenchymal–epithelial transition (*MET*) is crucial in the migration, proliferation, and invasion of tumour cells. Aberrant expression of *MET* and its ligand hepatocyte growth factor is associated with drug resistance in cancer therapy. Here, we found that *MET* was highly expressed in human CRPC tissues and overexpressed in DU145 and PC3 cells in a drug concentration‐dependent manner and is closely related to sensitivity to PARP inhibitors. Combining the PARP inhibitor olaparib with the MET inhibitor crizotinib synergistically inhibited CRPC cell growth both *in vivo* and *in vitro*. Further analysis of the underlying molecular mechanism underlying the MET suppression‐induced drug sensitivity revealed that olaparib and crizotinib could together downregulate the ATM/ATR signaling pathway, inducing apoptosis by inhibiting the phosphoinositide 3‐kinase/protein kinase B (PI3K/AKT) pathway, enhancing the olaparib‐induced antitumour effect in DU145 and PC3 cells. In conclusion, we demonstrated that MET inhibition enhances sensitivity of CRPC to PARP inhibitors by suppressing the ATM/ATR and PI3K/AKT pathways and provides a novel, targeted therapy regimen for CRPC.

## 1 INTRODUCTION

Prostate cancer (PC) is the second most common malignancy and a leading cause of cancer‐related death in men worldwide.^1^ The androgen receptor (AR) signaling axis is critical during all stages of PC genesis and plays a crucial role in cancer occurrence and progression. Hence, the mainstay therapy for PC is androgen deprivation therapy (ADT), which suppresses signaling of the AR by chemical or surgical castration. As the disease progresses, the patients eventually develop resistance toward ADT, leading to castration‐resistant PC (CRPC), at which stage the disease becomes fatal. In CRPC, existent treatment modalities provide limited effectiveness, and the prognosis is poor.^2^ Hence, improved and targeted therapeutic regimens are the need of the hour. On average, DNA in cells of the human body undergo tens of thousands of damages; these lesions can be classified as endogenous (e.g., reactive oxygen species and hydrolytic reactions) or exogenous (e.g., chemicals and radiations), which obstruct DNA replication and transcription, leading to cell cycle arrest or DNA lysis and collapse.^3^ Meanwhile, DNA damage response (DDR) is required to participate in DNA repair to protect the cell from the damage. The main executors of DDR are the poly adenosine diphosphate–ribose polymerase (PARP) family of inhibitors consisting of 17 members, of which PARP1 and PARP2 play a major role in the repair of single‐strand breaks (SSBs).^4^ The accumulation of SSBs eventually leads to double‐strand breaks (DSBs). Disruptive mutations in DNA damage repair genes such as *BRCA1*/*2*, *ATM*, and *RAD51* initiate the process of tumourigenesis. *BRCA1*/*2* and *ATM* are essential in the homologous recombination (HR) pathway, which plays an important role in DSBs. Tumours carry HR gene mutations that are sensitive to PARP inhibitors, leading to synthetic lethality, which is when deficiencies in the expression of two or more genes led to cell death.^5^, ^6^, ^7^ Up to 30% of patients with metastatic CRPC carry genomic alterations in the DDR, including in genes related to homologous recombination repair (HRR) deficiency. Among these genetic changes, the mutations in *BRCA2* are the most common.^8^ PARP inhibitor olaparib targets cancer cells with defects in the HRR, resulting in synthetic lethality. Given that olaparib is only suitable for mCRPC patients with deleterious or suspected deleterious germline or somatic HRR gene mutations, a study to improve the antitumour effect of drugs and to expand the population of drug applications so as to provide patients with more effective targeted treatment options is needed.

The proto‐oncogene mesenchymal–epithelial transition (*MET)* tyrosine kinase and its ligand hepatocyte growth factor (HGF) play an important role in the migration, proliferation, and invasion of tumour cells.^9^ Overexpression of the *MET* receptor is related to the poor prognosis of patients and drug resistance.^10^, ^11^ In addition, AR inhibition can significantly upregulate the expression level of *MET*, thereby playing a role in the transformation of androgen‐dependent PC to androgen‐independent PC.^12^, ^13^, ^14^ *MET* overexpression is observed in patients with advanced PC and is related to drug resistance.^15^, ^16^ It has been proposed that combined inhibition of MET and AR is more efficacious than using either drug alone.^17^ Moreover, MET phosphorylates PARP1 at pTyr907, which increases PARP1 enzyme activity and reduces the binding capacity of PARP inhibitors, resulting in drug resistance; while inhibition of MET enhances the antitumour effect of PARP inhibitors,^18^ but whether targeting MET also can improve the antitumour effect of PARP inhibitors in PC is currently unknown. In this study, we found that MET is highly expressed in PC cell lines exposed to olaparib in a concentration‐dependent manner, and its expression is closely related to sensitivity to olaparib. We also examined whether the PARP inhibitor olaparib, used either alone or in combination with the MET inhibitor crizotinib, could effectively be used to treat PC.

## 2 MATERIALS AND METHODS

### 2.1 Cell culture and inhibitors

Human PC cell lines (DU145, PC3, LNCaP, and 22RV1) were purchased from Procell. PC3, LNCaP, and 22RV1 cells were cultured in RPMI 1640 medium (Sevenbio) with 10% fetal bovine serum (FBS; Gibco), and DU145 cells were cultured in Dulbecco's modified eagle medium (Sevenbio) supplemented with 10% FBS. All the cell lines were cultured in a cell incubator with 5% CO_2_, and the temperature was maintained at 37°C. Olaparib and crizotinib were obtained from Selleck, SC79 (HY‐18749) was purchased from MedChemExpress.

### 2.2 Antibodies

Antibodies against MET (ab51067), p‐MET (ab68141), cleaved PARP1 (ab32064), PI3K (ab191606), p‐PI3K (ab182651), p‐ATM (ab81292), p‐ATR (ab178407), and AKT (ab8805) were purchased from Abcam (Cambridge, England). Antibodies against GAPDH (6004–1‐Ig), BCL‐2 (60178–1‐Ig), ATM (27156–1‐AP), ATR (19787–1‐AP), IgG (H + L) (SA00013‐4), and RAD51 (14961–1‐AP) were purchased from Proteintech (Wuhan, China). Antibodies against γH2AX (#9718), Ki67 (#12202), and cleaved caspase‐3 (#9664) were purchased from Cell Signaling Technology.

### 2.3 Western blot analysis

Protein was extracted from harvested cells using radioimmunoprecipitation assay buffer (KeyGEN BioTECH) supplemented with the protease inhibitor phenylmethylsulfonyl fluoride (PMSF; KeyGEN BioTECH) and phosphatase inhibitor cocktails (KeyGEN BioTECH). All the protein concentrations were detected using BCA (bicinchoninic acid) assay kit (Thermo Fisher Scientific). The same amounts of the extracted protein samples were supplemented with loading buffer (KeyGEN BioTECH) and separated in 4%–12% sodium dodecyl sulphate–polyacrylamide gel electrophoresis (SDS‐PAGE) gel (Sevenbio), and then transferred to a nitrocellulose membrane (Millipore, Missouri, USA). After incubation of the membranes with antibodies, the protein on the membranes was detected on an enhanced chemiluminescence (ECL) Western blotting substrate (Tanon, China). Protein membranes were analyzed by Image J software. All the experiments were conducted at least by three independent researchers.

### 2.4 Quantitative real‐time PCR

Total RNA in PC cells was isolated by TRIzol (TaKaRa Bio) reagent and reverse transcribed to Complementary DNA (cDNA) using the PrimeScript reverse transcription (RT) reagent kit (TaKaRa Bio, Dalian, China) following the manufacturer's instructions. Real‐time PCR (qPCR) was conducted with QuantiTect SYBR Green PCR kit (TaKaRa Bio) with the Stratagene MX3000P qPCR system (Agilent). 2^−ΔΔCt^ method was performed to calculate the relative quantities of MET and GAPDH mRNA expression. The qPCR primers sequences of MET and GAPDH (synthesized by Genepharma, Suzhou, China) were follows:

GAPDH: 5′‐ CATGAGAAGTATGACAACAGCCT‐3′ (forward).

5′‐AGTCCTTCCACGATACCAAAGT‐3′ (reverse).

MET: 5′‐ TCCAGGCAGTGCAGCATGTA‐3′ (forward).

5′‐TCAAGGATTTCACAGCACAGTGA‐3′ (reverse).

### 2.5 Colony formation assay

For the colony formation assay, cells were plated at a uniform concentration of approximately 700 cells/well into six‐well plates and then incubated for three weeks in medium containing a single drug or a combination of drugs, and the medium was refreshed every 2 days. To detect colony formation, the cultured cells were then fixed independents with paraformaldehyde, stained with crystal violet, dried, and counted.

### 2.6 Transwell migration assay

For the cell migration assay, the cells were plated into Transwell chambers (Corning, New York, USA) at a concentration of 2×10^4^ per well, incubated with 250 μl of serum‐free medium containing one drug or a combination of drugs. After 72 h of incubation, the cells were fixed with paraformaldehyde, stained with crystal violet, dried, and imaged. The results were analyzed using Image J software.

### 2.7 Cell viability assay

Cell Counting Kit‐8 (CCK‐8; KeyGEN BioTECH) was used to determine cell viability and growth. The cells were plated into the wells of 96‐well plates at a concentration of approximately 4 × 10^3^ cells per well, and then incubated in medium containing one or a combination of drugs. After 72 h of incubation, the medium was supplemented with 10% CCK‐8 and then incubated again for 2 h. The experimental results (optical density) were analyzed on a Multiskan™ FC Microplate Reader (Thermo Fisher Scientific) at 450 nm.

### 2.8 Silencing and overexpressing of MET

PC cells (5 × 10^4^ per well) were plated into the wells of six‐well plates and transfected with small‐interfering RNA (siRNA) sequences targeting MET (GenePharma; siMET 1: 5′‐GUGCCACU AACUACAUUUATT‐3′ and siMET 2: 5′‐GCUGGUGGCACUUUACUUATT‐3′ or nontargeting siRNA) using RNAi‐Mate (GenePharma) according to the manufacturer's protocol. The MET‐overexpression lentivirus (OE‐MET) was purchased from (GenePharma). LNCaP and 22RV1 cells were transduced with the lentivirus in the presence of polybrene (8 μg/ml), after that, those successfully transfected cells were selected with puromycin (2 μg/ml).

### 2.9 Drug combination index

The drug combination index (CI) value was measured using CompuSyn software. CI value less than 1 indicates the presence of a synergistic effect between the two drugs, and the smaller the value, the stronger is the synergistic effect; a CI value equal to 1 indicates that there is only an additive effect between the drugs; and a CI value greater than 1 indicates that the drugs are antagonistic.^19^

### 2.10 Immunohistochemical assay

Immunohistochemical (IHC) assays were performed to validate the expression of γH2AX, Ki67, cleaved caspase‐3, and RAD51 antibodies in mouse subcutaneous tumour tissues. Subcutaneous tumour tissues were deparaffinized and rehydrated, and then supplemented with 3% hydrogen peroxide–methanol solution. After 30 minutes, preincubation was done in 5% bovine serum albumin (BSA; Gibco). To avoid nonspecific staining, the subcutaneous tumour tissues were probed with antibodies of γH2AX (1:100), Ki67 (1:100), cleaved caspase‐3 (1:100), and RAD51 (1:100) at 4°C overnight. Next, the tissue sections were supplemented with biotinylated secondary antibodies for 20 minutes and then stained using a diaminobenzidine kit (Lab Vision). The scores of all the IHC tissue sections were evaluated by two independent researchers. Counterstaining was evaluated using Image J software.

### 2.11 Immunofluorescence

PC cells (DU145 and PC3) were plated at a concentration of 8 × 10^3^ per well into 24‐well plates to quantify γH2AX. After 48 h of treatment, the cells were fixed with 4% paraformaldehyde (KeyGEN BioTECH) for 25 min and then permeabilized with 0.25% Triton X‐100 (Meilunbio) for 10 min. The cells were blocked in 5% BSA (Sigma) for 60 min at room temperature and then supplemented with primary antibody γH2AX (1:400) overnight at 4°C. Subsequently, the PC cells were counterstained by Alexa Fluor 594 (1:1000; Proteintech) for 1 h and then counterstained by DAPI (1:1000, KeyGEN BioTECH) for 15 min at room temperature in a dark place. Images were acquired using a fluorescence microscope (Nikon TE200, Japan). Counterstaining was evaluated using Image J software.

### 2.12 Mouse xenograft models

DU145 cells (2 × 10^6^ mixed with 100 μl phosphate‐buffered saline) were inoculated into the flank of male BALB/c nude mice. After 10 days, when the subcutaneous tumour volumes reached 50 mm^3^, the nude mice were injected intraperitoneally with the PARP inhibitor olaparib (40 mg/kg) and the MET inhibitor crizotinib (5 mg/kg), either alone or in combination for 5 days per week for a total of 4 weeks. The tumour volume was measured every 4 days.

All animals are handled strictly in accordance with the recommendations formulated by the Animal Research and Care Committee of Dalian Medical University, and the study was performed according to the guidelines for animal experimentation of Dalian Medical University.

### 2.13 Statistical analysis

All experimental data were captured using GraphPad Prism 8.0.2 software and the statistical differences were analyzed using SPSS Version 22.0 software. Statistical significance was defined when a *p*‐value was <0.05.

## 3 RESULTS

### 3.1 MET is overexpressed in olaparib‐treated PC cells in a concentration‐dependent manner

**FIGURE 1:**
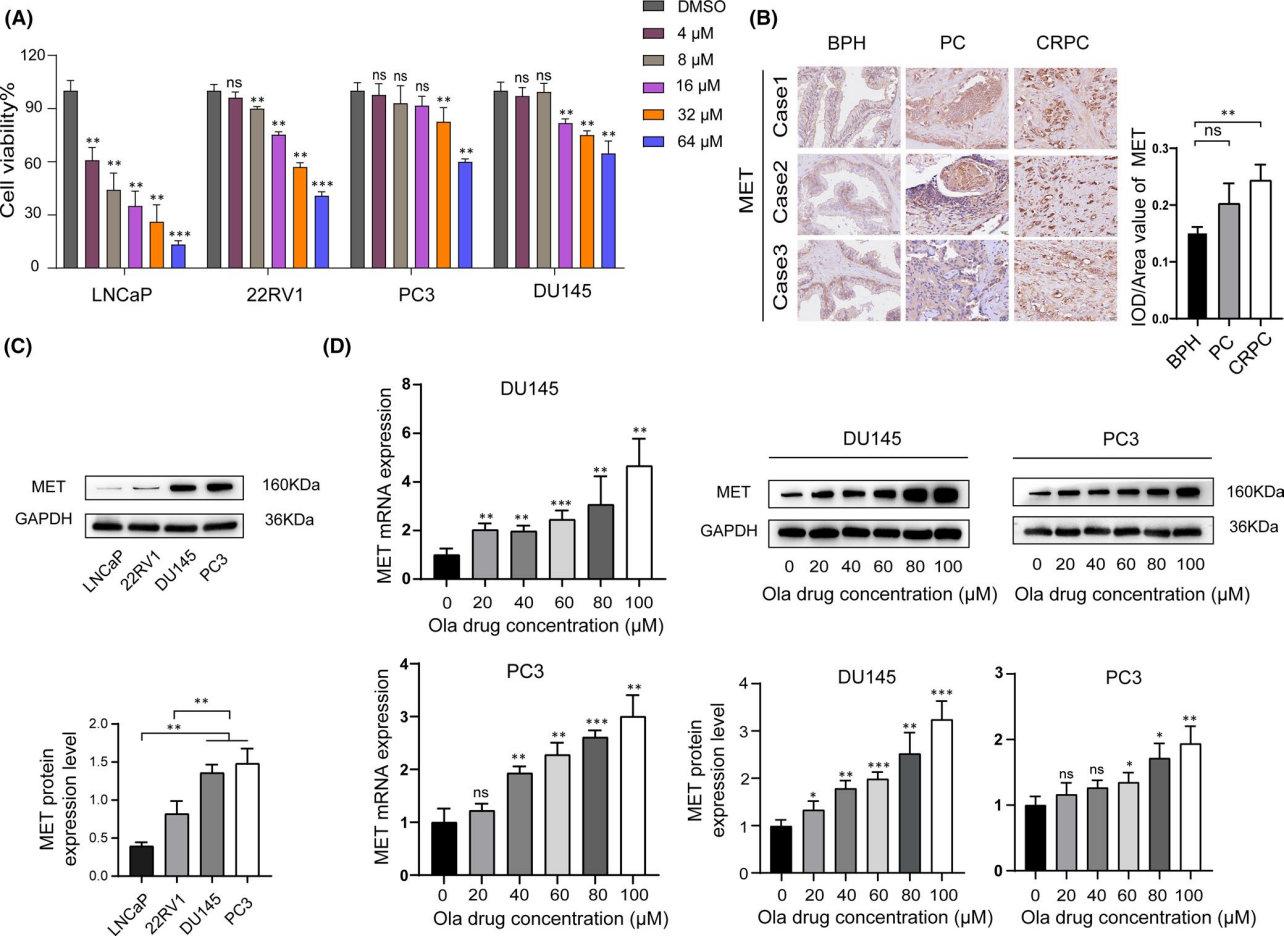
MET is overexpressed in a concentration‐dependent manner in olaparib‐treated prostate cancer (PC) cells. (A) PC cells (LNCaP, 22RV1, DU145, and PC3) were treated with increasing concentrations (4–64 μM) of olaparib or dimethyl sulphoxide for 72 h. (B) Representative immunohistochemical analysis of MET protein expression in human BPH, hormone‐sensitive prostate cancer, and CRPC tissues. (C) Western blot analysis of the expression of MET in PC cells (LNCaP, 22RV1, DU145, and PC3). (D) MET mRNA and protein expression increased in olaparib‐treated PC cells (DU145 and PC3) in a concentration‐dependent manner. Statistically significant differences were assessed by Student's *t* test in three independent experiments. *p < 0.05, **p < 0.01, ***p < 0.001. ns = no statistical difference

To evaluate the drug sensitivity of olaparib in PC cell lines, the cells (LNCaP, 22RV1, DU145, and PC3) were exposed to olaparib with increasing concentrations (4–64 μM) for 72 h. The sensitivity of the cells to olaparib was detected by CCK‐8 after 72 h of treatment (Figure 1A). The results showed that PC3 and DU145 cells were relatively insensitive to olaparib compared with LNCaP and 22RV1 cells, and the half‐maximal inhibitory concentration (IC_50_) of olaparib in DU145 was approximately 13.5 times that in LNCaP cells (Table 1). The proto‐oncogene *MET*, which is highly expressed in CRPC, is known to be associated with cancer occurrence, progression, and treatment resistance.^20^ To observe the expression of *MET* in prostate tissue, we collected human benign prostatic hyperplasia (BPH), hormone‐sensitive PC, and CRPC tissues. As indicated, MET protein was overexpressed in CRPC (Figure 1C). Next, we detected MET expression in the PC cell lines by Western blot analysis, and found that similar to that in CRPC, MET protein was highly expressed in PC3 and DU145 cells (Figure 1B). Moreover, the levels of MET mRNA and protein in DU145 and PC3 cells treated with increasing concentrations of olaparib showed significant upregulation (Figure 1D). These results indicate that MET is closely related to sensitivity of PC cell lines to olaparib.

**TABLE 1:** IC_50_ of olaparib in prostate cancer cells

Cell	LNCaP	22RV1	DU145	PC3
IC_50_	5.43 μM	22.83 μM	73.20 μM	64 μM

Abbreviation: IC_50_, half‐maximal inhibitory concentration

### 3.2 Targeting MET induces olaparib sensitivity in vitro

**FIGURE 2:**
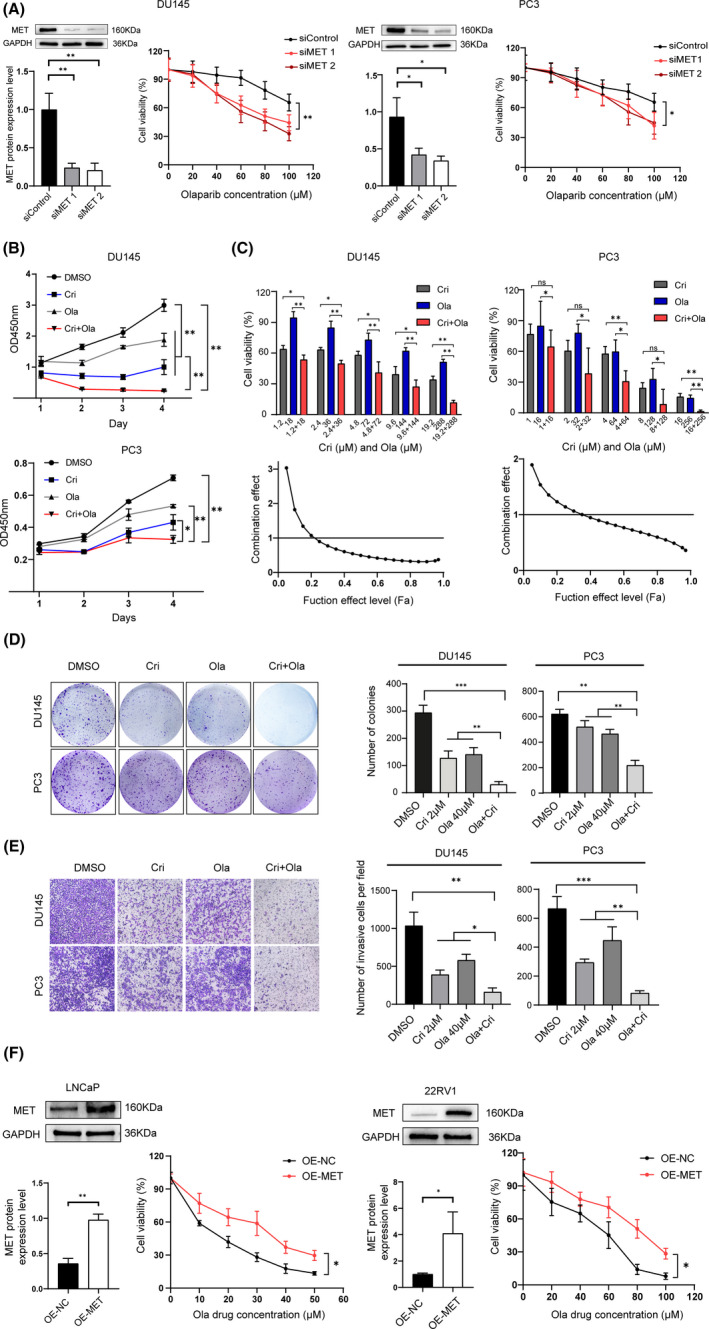
MET suppression induces olaparib sensitivity *in vitro*. (A) Western blot shows siRNAs effectively silencing MET in PC cells. Viability of DU145 and PC3 cells transfected with control or MET siRNAs and treated with dimethyl sulphoxide or olaparib for 3 days. (B) Cell viability of the combination treatment of Cri (MET inhibitor crizotinib) and Ola (PARP inhibitor olaparib) or monotherapy in DU145 and PC3 cells for different durations (0–96 h). (C) Synergistic effects (combination index, CI) of Cri and Ola in PC cells (DU145 and PC3) was measured using CompuSyn software. (D) Clonogenicity of DU145 and PC3 cells were performed by clonogenic formation assay after treatment with Cri (2 μM) and Ola (64 μM), monotherapy or dimethyl sulphoxide for 14 days. E, Migration assays were performed in the presence of Cri (2 μM) and Ola (40 μM), Cri or Ola monotherapy, or dimethyl sulphoxide in DU145 and PC3 cells for 3 days. F, Western blot shows the overexpression of exogenous MET in prostate cancer cell lines LNCaP and 22RV1, then examining the drug resistance following in the presence of different drug concentrations. Statistically significant differences were assessed by Student's *t* test in three independent experiments. *p < 0.05, **p < 0.01, ***p < 0.001. ns =no statistical difference

To evaluate whether MET mediates tumour sensitivity to olaparib, we used siRNA to silence MET expression and examined the growth of DU145 and PC3 cells in the presence of olaparib. MET silencing enhanced the inhibition of olaparib in DU145 and PC3 cells by decreasing cell viability (Figure 2A). Furthermore, we investigated whether crizotinib could also influence the tumour response to olaparib in PC cells. As indicated, crizotinib rendered the DU145 and PC3 cells more sensitive to olaparib by suppressing cell proliferation (Figure 2B). In addition, the CI values indicated that treatment with the combination of olaparib and crizotinib could synergistically inhibit the growth of DU145 and PC3 PC cells (Figure 2C). Moreover, we observed significant synergistic antineoplastic effects on colony formation in the Transwell migration assay for the olaparib–crizotinib combination compared with monotherapy with either drug alone (Figure 2DandE). Moreover, the overexpression of exogenous MET in prostate cancer cell lines LNCaP and 22RV1 resulted in drug resistance (Figure 2F). These results demonstrate that MET inhibition enhances the antineoplastic effect of olaparib in PC cells and that the combination of crizotinib and olaparib have a strong synergistic effect.

### 3.3 MET suppression downregulates ATM/ATR pathway and enhances sensitivity to PARP inhibition

**FIGURE 3:**
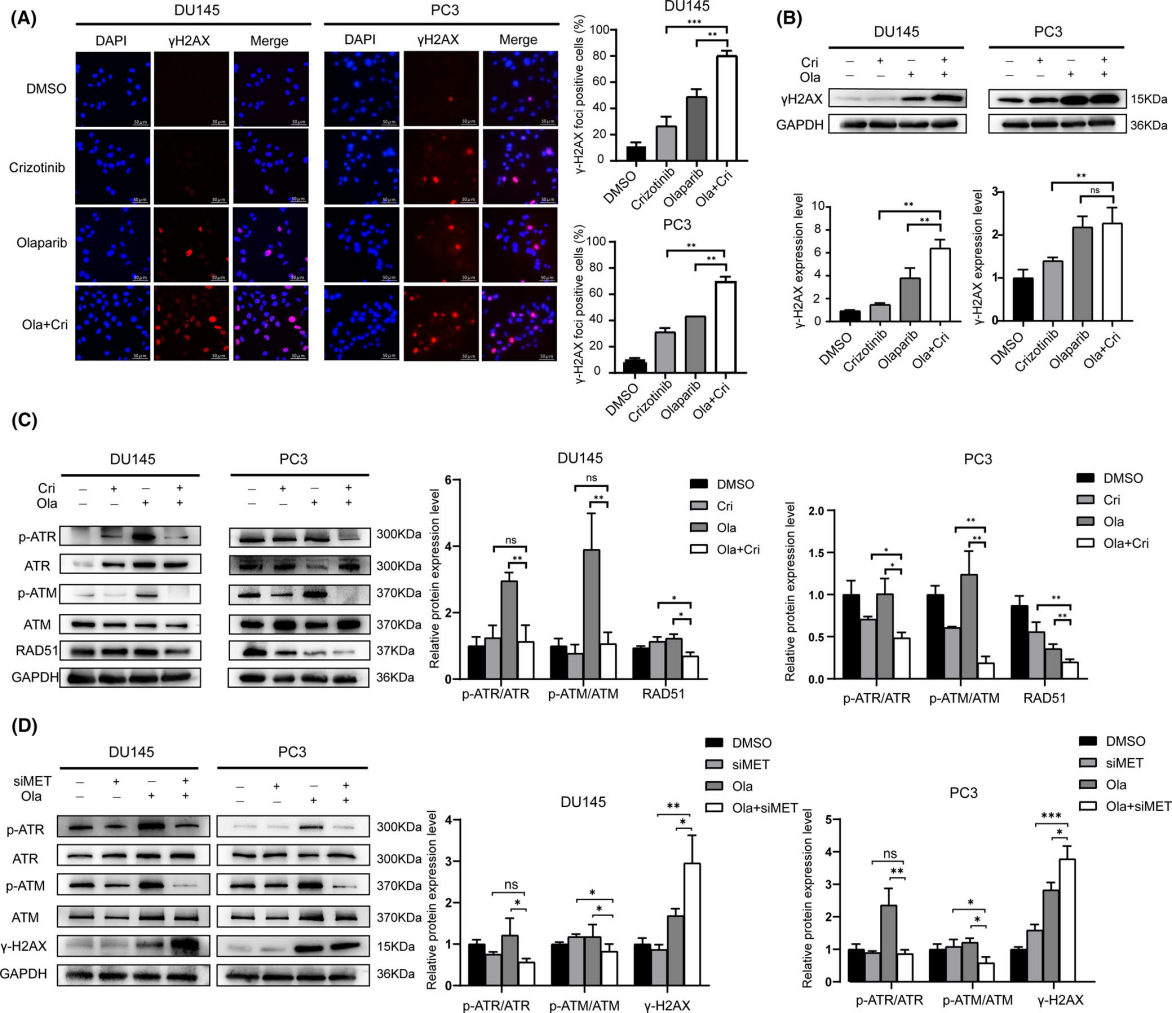
γH2AX staining and ATM/ATR pathway downregulation following MET suppression and enhancing sensitivity to olaparib. (A) Representative immunofluorescent staining of γH2AX (red) and DAPI (blue) in DU145 and PC3 cells in the presence of Cri ((MET inhibitor crizotinib; 4 μM) and Ola (PARP inhibitor olaparib; 64 μM), Cri or Ola monotherapy, or dimethyl sulphoxide for 3 days. Scale bar, 50 μm. More than five foci per nucleus were considered as positive cells. (B) Western blot analysis of γH2AX expression in DU145 and PC3 cells. (C‐D) Western blot analysis of p‐ATM/ATM, p‐ATR/ATR, and RAD51 expression in DU145 and PC3 cells. Statistically significant differences were assessed by Student's *t* test in three independent experiments. *p < 0.05, **p < 0.01, ***p < 0.001. ns = no statistical difference

HRR‐deficient cells that are treated with PARP inhibitors trigger synthetic lethality, causing irreversible disruption of chromosomal construction, cell cycle arrest, and ultimately cell death.^21^ Therefore, HRR deficiency can enhance sensitivity to PARP inhibition. To examine whether MET suppression renders PC cells sensitive to olaparib by inducing HRR deficiency, we assessed the expression level of the DNA damage marker *γH2AX* in PC cell lines (DU145 and PC3) treated with either olaparib or crizotinib alone or in combination. Results of the immunofluorescence analysis revealed a significant increase in the accumulation of γH2AX foci in cells that were subjected to the combined treatment of olaparib and crizotinib than in those that received monotherapy or vehicle treatment (Figure 3A). Similarly, results of the Western blot analysis showed similar results that γH2AX expression markedly increased in the DU145 and PC3 cells treated with olaparib and crizotinib combination compared with cells treated with monotherapy or vehicle (Figure 3B).

The mechanism of action of DDR includes numerous coordinated checkpoints and repair paths that regulate the checkpoints and apoptosis or repair DNA damages to maintain DNA integrity in human body cells. The primary proteins of the signal transduction response are ATM and ATR.^22^, ^23^ In addition, it has been proposed that MET could phosphorylate the RAD51 protein, which is the key protein in HRR, and that inhibition of MET is accompanied by downregulation of RAD51 phosphorylation.^24^ Thus, we tested the expression levels of *ATM*, *ATR*, and *RAD51*, the central DDR proteins, in DU145 and PC3 cells following treatment with olaparib or crizotinib alone or a combination of the two. The results show that the expression levels of *p*‐*ATM*/*ATM*, *p*‐*ATR*/*ATR*, and *RAD51* decreased in the combination therapy compared with monotherapy or vehicle (Figure 3CandD). Moreover, MET silencing then treated with the combination of crizotinib and olaparib in PC cells also downregulated the expression of *p*‐*ATM*/*ATM*, *p*‐*ATR*/*ATR* (Figure 3E). Our results indicated that the MET inhibitor downregulates the expression of *p*‐*ATM*/*ATM*, *p*‐*ATR*/*ATR*, and *RAD51* and enhances sensitivity to PARP inhibition.

### 3.4 Cotargeting PARP and MET induces apoptosis of PC cells by inhibiting the PI3K/AKT pathway

**FIGURE 4:**
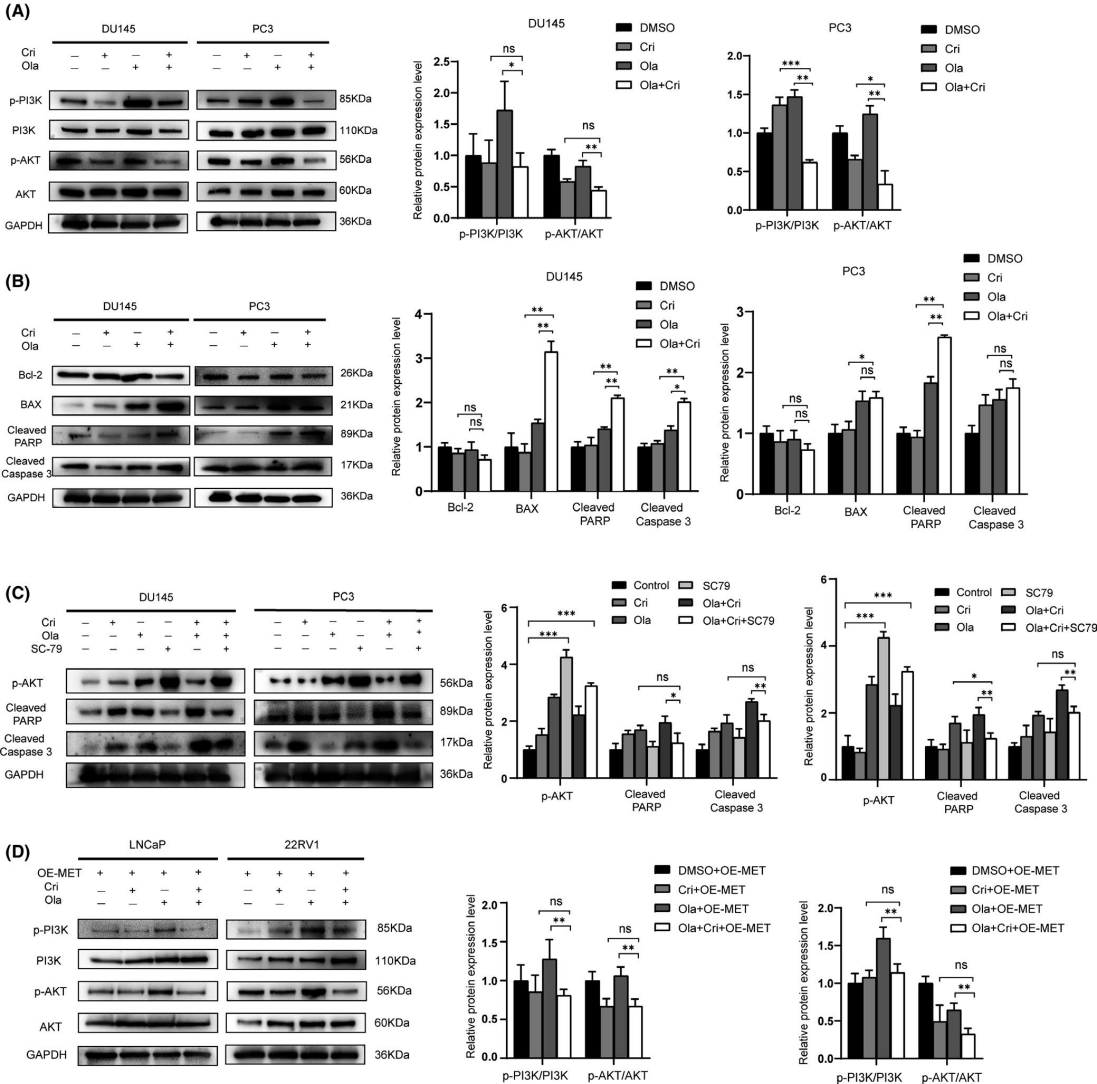
Combined treatment of olaparib with crizotinib induces apoptosis of the PC cells by inhibiting the PI3K/AKT pathway. (A) Western blot analysis of the expression of p‐PI3K/PI3K and p‐AKT/AKT in DU145 and PC3 cells. (B) Western blot analysis of the expression of BCL‐2, BAX, cleaved PARP, and cleaved caspase 3 in DU145 and PC3 cells. (C) Cells were pretreated with or without 2 μg/ml SC79 and then treated with Cri (4 μM) and Ola (64 μM), Cri or Ola monotherapy, or dimethyl sulphoxide for 3 days, then Western blot analysis of the expression of p‐AKT, cleaved PARP, and cleaved caspase 3. (D) The OE‐MET of LNCaP and 22RV1 in the presence of Cri (4 μM) and Ola (64 μM), Cri or Ola monotherapy, or dimethyl sulphoxide for 3 days, then Western blot analysis of the expression of PI3K/AKT pathway. Statistically significant differences were assayed by Student's *t* test in three independent experiments. *p < 0.05, **p < 0.01, ***p < 0.001. ns = no statistical difference

The PI3K/AKT pathway is a crucial sensor of genomic integrity. PI3K signaling promotes DNA double‐strand repair by interacting with the HRR complex, and PI3K suppression enhances the antitumor effect of PARP inhibitors.^25^, ^26^, ^27^ In addition, MET/HGF signaling protects tumour cells from DNA damage by activating the PI3K/AKT pathway.^28^ Therefore, we tested whether the inhibition of MET could downregulate the expression of PI3K pathway. As expected, crizotinib suppressed the phosphorylation of PI3K and AKT, which resulted in HRR deficiency, and finally enhanced the antineoplastic effects in PC cells (Figure 4A). Given that cleaved PARP and cleaved caspase 3 are the cleaved version of PARP and caspase 3 proteins, respectively, their activation plays an important role in apoptosis and can be used as apoptosis markers.^29^ Therefore, we examined the expression levels of cleaved PARP and cleaved caspase 3 in the PC cell lines DU145 and PC3 following treatment with olaparib or crizotinib monotherapy or a combination of the two. The results showed that in both DU145 and PC3 cell lines, cleaved PARP and cleaved caspase 3 protein levels were higher in the combination treatment group than in both the monotherapy group, indicating that combined therapy with these two drugs induced more apoptosis. In addition, we tested the antiapoptotic protein BCL‐2 and the proapoptotic protein BAX. We observed an increase in BAX protein expression and a decrease in BCL‐2 protein expression in the DU145 and PC3 cell lines treated with combination therapy compared with monotherapy or vehicle (Figure 4B). In order to confirm that the drug resistance mediated by MET is due to PI3K/AKT over‐activation. We used SC79, an AKT activator, after the combined treatment of crizotinib and olaparib in PC cells. As indicated, SC79 could suppress the expression of cleaved PARP and cleaved caspase 3 and decreased cell apoptosis (Figure 4C). Furthermore, we conducted the overexpression of exogenous MET in prostate cancer cell lines LNCaP and 22RV1 to reach olaparib‐resistance states, and following treatment with olaparib or crizotinib alone or a combination of the two, then detected the expression of PI3K/AKT by western blot. As indicated, the combined treatment in LNCaP and 22RV1 could suppress the PI3K/AKT pathway (Figure 4D). Our results showed that the combined inhibition of PARP and MET induces apoptosis of DU145 and PC3 cells by inhibiting the PI3K/AKT pathway.

### 3.5 Olaparib and crizotinib synergistically inhibit the growth of subcutaneous tumours in vivo

**FIGURE 5:**
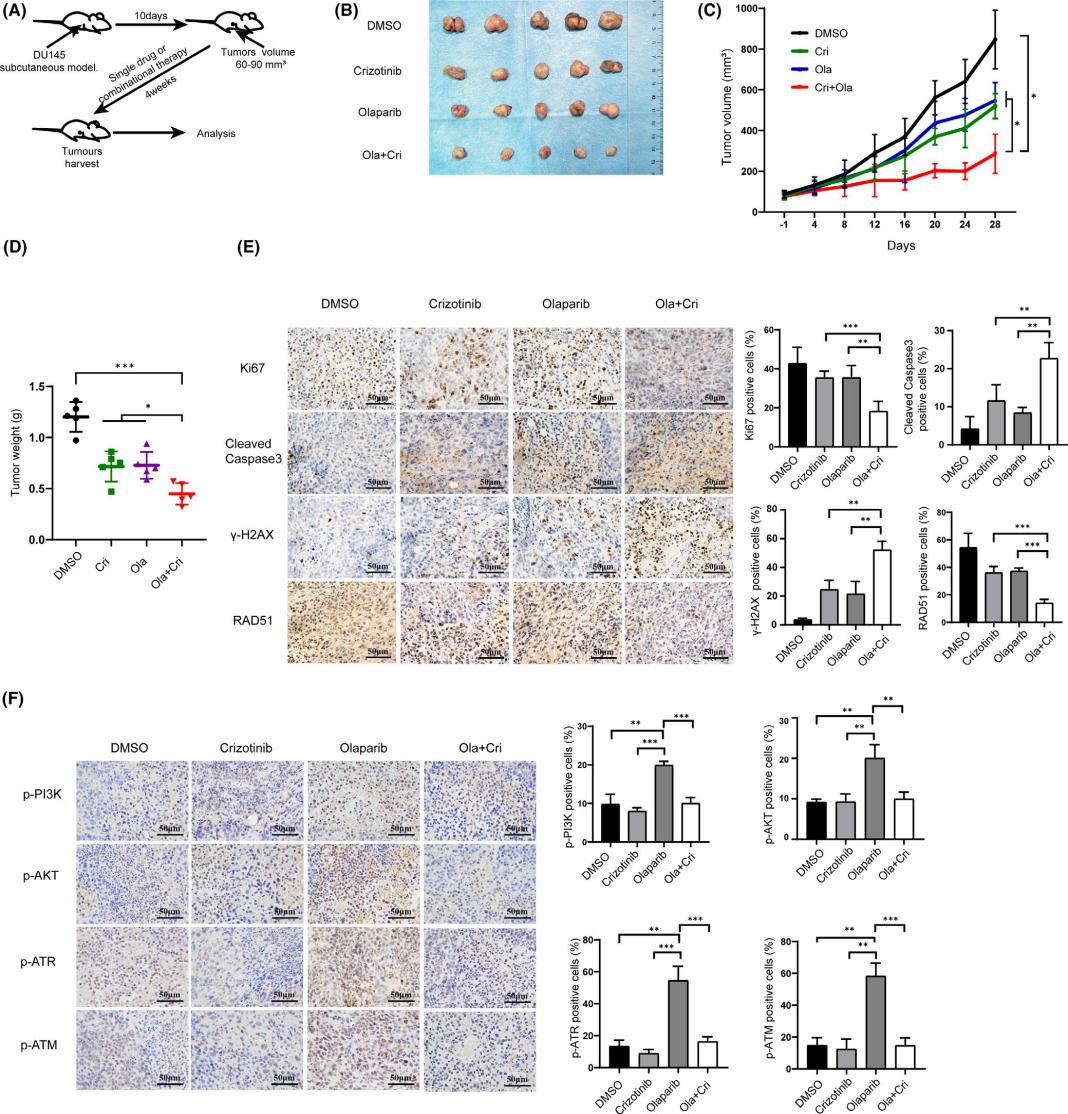
Olaparib and crizotinib synergistically inhibit the growth of subcutaneous tumours *in vivo*. Once DU145 subcutaneous tumour reached 50 mm^3^, mice were injected intraperitoneally with the olaparib (Ola, 40 mg/kg) and crizotinib (Cri, 5 mg/kg), either alone or in combination for 4 weeks (5 day per week). (A) Brief experiment process diagram. (B–D) Curves of tumour volume growth, representative gross images of tumour sizes, and tumour weight after treating with different groups as indicated. (E‐F) The representative immunohistochemical analysis of the expression of Ki67, RAD51, cleaved caspase 3, γH2AX, p‐PI3K, p‐AKT, p‐ATR and p‐ATM proteins in DU145 xenografted tumour cells after treating with different groups as indicated. Statistically significant differences were assessed by one‐way analysis of variance and Student's *t* test. *p < 0.05, **p < 0.01, ***p < 0.001. ns = no statistical difference

To further confirm our *in vitro* experimental results, we established DU145 subcutaneous tumour models *in vivo*. As indicated, the combined treatment of olaparib and crizotinib significantly slowed down the growth of subcutaneous tumours in mice compared with those treated with olaparib or crizotinib alone (Figure 5A–C). In addition, we removed the subcutaneous tumours from the nude mice and weighed it after the completion of the experiment. The tumours weighed significantly lower in mice treated with the combination therapy compared with those in mice in the monotherapy and control groups (Figure 5D). An analysis of the subcutaneous tumours showed substantially reduced Ki67 staining and RAD51 protein abundance but increased cleaved caspase 3 and γH2AX protein levels in mice in the combination treatment group compared with mice in the monotherapy and control groups (Figure 5E). In addition, the phosphorylation expression levels of PI3K, AKT, ATR and ATM decreased in the combination therapy compared with monotherapy or vehicle in subcutaneous tumours (Figure 5F). Therefore, our results suggested that the combination of olaparib and crizotinib could synergistically inhibit the growth of subcutaneous PC tumours *in vivo*.

## 4 DISCUSSION

The amassing of genetic and epigenetic aberrations leads to the initiation of carcinogenesis in PC. Multiple reasons such as altered transcription of AR signaling, PI3K signaling, and DNA repair defects can be attributed to PC carcinogenesis.^30^ The AR signaling axis, which is a PC characteristic, plays a key role in cancer progression, with ADT being the mainstay of therapy.^31^ However, ADT fails as an effective treatment strategy in advanced PC, and hence, a targeted therapy that formulates a personalized treatment option based on the patient's genetic characteristics is one of the important methods of cancer treatment. Up to 30% of advanced CRPC patients harbor germline or somatic HRR gene deficiencies that render these cells sensitive to PARP inhibitors. According to the Trial of PARP Inhibition in Prostate Cancer (TOPARP)‐A and TOPARP‐B, the PARP inhibitors have achieved great progress in the treatment of mCRPC patients who carry HRR deficiency.^32^, ^33^ But in patients who do not carry HRR deficiency, the use of olaparib in the treatment of PC is limited.^32^ Furthermore, as with other targeted therapies, the development of drug resistance is inevitable, which hinders the clinical application of PARP inhibitors in mCRPC patients. Whether the drug resistance mechanism is preclinical or clinically reported, the reverse mutations of the *BRCA1*/*2* gene are the main reasons that lead to the restoration of HR and ultimately the resistance of PARP inhibitors observed in PC.^34^ Application of PARP inhibitors in the clinical treatment of PC will result in the gradual emergence of resistance to these drugs. Although PARP inhibition offers a potentially effective option for cancers that harbor a disruptive mutation in HRR genes, drug resistance proves to be a “stumbling block” in the successful management of cancer.

Currently, four main mechanisms by which cancer cells exhibit resistance to PARP inhibitors have been reported. First, the *Abcb1a* and *Abcb1b* genes, members of the ATP‐binding cassette, encoding the multidrug resistance protein (MDR1 or P‐gp) involved in resistance of BRCA1/2‐deficient breast and ovarian cancer patients to PARP inhibitors, are upregulated.^35^, ^36^ Consequently, the inhibition of P‐gp (*Abcb1*) could resensitize olaparib‐resistant cell lines.^37^ Second, PARP inhibitors not only directly target the enzymatic action of PARP proteins but also trap PARP enzymes at the DNA damage site to form PARP–DNA cytotoxic complexes. PARP1 is the main protein responsible for the PARylation of DNA damage.^38^ Thus, studies have shown that the loss‐of‐function alterations of PARP1 led to a 100‐fold higher olaparib resistance than its wild‐type counterpart, and elevating the PARP1 expression may make tumours more sensitive to PARP inhibitors.^39^ Third, PARP inhibition and HRR defects are the two complementary hallmarks of inducing synthetic lethality by PARP inhibitors in cancer treatment. Disruptive mutations in HRR have been shown to enhance the sensitivity of BRCA1/2‐mutated cancer cells to PARP inhibitors. Conversely, secondary mutations in these cells may reactivate the functions of HRR and result in the resistance of PARP inhibitors.^40^, ^41^ Fourth, the PARP inhibitors require abundant lethal DNA damage to initiate apoptosis in BRCA‐mutated cancer cells. Thus, the reduction of damage formation, especially the reduction of fatal damage, is an important reason for the resistance of PARP inhibitors. The replication fork is precise and complex and hence, replication fork pausing can result due to various internal and external factors. DNA damage is one of the reasons that causes fork pausing, and stalled replication forks are protected by BRCA1/2.^42^, ^43^ These protections are eliminated in BRCA‐mutated cancer cells, which result in the degradation of the replication fork. Thus, stabilizing the stalled replication fork is another mechanism that induces resistance to PARP inhibitors.^42^, ^44^ Studies have also shown that the loss of PTIP/MLL3/MLL4 complexes can stabilize the replication fork by protecting BRCA1/2‐mutated cells from DNA damage induced by the degradation of nascent DNA strands.^45^ Also, Clements et al. identified that the loss of transcriptional repressor E2F7 caused resistance to PARP inhibitors and cisplatin in BRCA2‐deficient cells, and the reason was attributed to E2F7 depletion by upregulating the expression of *RAD51*, promoting HRR and stability of the replication fork.^46^ In addition, the loss of transcriptional repressor EZH2/MUS81 axis, which is associated with stalled replication fork collapse, also results in resistance of BRCA2‐deficient cells to PARP inhibitors.^47^, ^48^ Therefore, to improve the antitumour effect of PARP inhibitors and to overcome the emergence of drug resistance, patients should be provided with more effective and targeted treatment options.

*MET*, one of the receptor tyrosine kinases, is related to embryogenesis, organofaction, tumourigenesis, and cancer metastasis. Moreover, the aberrant expression of *MET* and its ligand, HGF, is associated with poor prognosis and drug resistance in cancers.^49^ The MET signaling pathway can be activated by multiple molecules, plexins, integrins, EGFR, and ERBB2, and the crosstalk between those pathways may contribute to cancer progression and drug resistance.^50^, ^51^ Some studies have shown that the activation of MET signaling drove the resistance to EGFR inhibitors in lung cancer.^52^, ^53^ Moreover, it has been proposed that MET inhibition enhances the antitumour effect of PARP inhibitors in multiple cancers.^54^, ^55^ In our study, we found that MET was highly expressed in PC cell lines treated with olaparib in a concentration‐dependent manner and that it was closely related to the sensitivity of olaparib. Furthermore, combining olaparib with crizotinib resulted in a synergistic inhibition of growth in PC tumours both *in vivo* and *in vitro*. To further elucidate the molecular mechanism underlying MET suppression due to drug sensitivity, we examined the HR relative genes *ATM*, *ATR*, and *RAD51*. The results showed that a combination of olaparib and crizotinib could downregulate the ATM/ATR/RAD51 signaling pathway and induce HRR deficiency, thereby enhancing the olaparib‐induced antitumour effect in PC DU145 and PC3 cells. Moreover, studies show that the PI3K pathway is a crucial sensor of genomic integrity and the PI3K downstream gene *AKT* is activated when exposed to PARP inhibitors, and these changes limit the efficacy of PARP inhibitors in the treatment of cancers. In addition, some studies found that the aberrant expression and activation of *MET* result in the activation of the ERBB3/PI3K/AKT pathway, which is associated with the resistance of EGFR inhibitors in lung cancer, and this resistance can be reversed by combining MET inhibitors and EGFR inhibitors. In the current study, we observed that MET inhibition downregulated the PI3K/AKT signaling pathway, induced apoptosis, and enhanced the drug sensitivity of olaparib in PC. In conclusion, our study demonstrates that MET inhibition enhances the efficacy of PARP inhibitors in PC and provides a novel, targeted, therapy regimen for the management of advanced PC.

## CONFLICT OF INTEREST

The authors declare no conflict of interest.

## AUTHOR CONTRIBUTIONS

**Sihai Zhou:** Data curation (lead); Formal analysis (lead); Investigation (lead); Methodology (lead); Writing‐original draft (lead); Writing‐review & editing (lead). **Zhihong Dai:** Data curation (lead); Formal analysis (lead); Investigation (lead); Methodology (lead); Writing‐original draft (lead); Writing‐review & editing (lead). **Liang Wang:** Data curation (equal); Formal analysis (equal); Methodology (equal); Writing‐original draft (equal); Writing‐review & editing (equal). **Liqin Yang:** Data curation (equal); Investigation (equal); Writing‐original draft (equal). **Xiang Gao:** Data curation (equal); Formal analysis (equal); Investigation (equal); Methodology (equal); Writing‐original draft (equal); Writing‐review & editing (equal). **Zhenwei Wang:** Data curation (equal); Formal analysis (equal); Investigation (equal); Methodology (equal). **Qi Wang:** Conceptualization (equal); Funding acquisition (equal); Project administration (equal); Supervision (equal); Writing‐original draft (equal); Writing‐review & editing (equal). **Zhiyu Liu:** Conceptualization (lead); Funding acquisition (lead); Project administration (lead); Supervision (lead); Writing‐original draft (lead); Writing‐review & editing (lead).

## Data Availability

All data supporting or described during this study are available in this published article. Those data used to support the findings of this study are available from the corresponding author upon request.
